# Of Men Not Mice: Bactericidal/Permeability-Increasing Protein Expressed in Human Macrophages Acts as a Phagocytic Receptor and Modulates Entry and Replication of Gram-Negative Bacteria

**DOI:** 10.3389/fimmu.2016.00455

**Published:** 2016-10-24

**Authors:** Arjun Balakrishnan, Markus Schnare, Dipshikha Chakravortty

**Affiliations:** ^1^Department of Microbiology and Cell Biology, Indian Institute of Science, Bangalore, India; ^2^Institute for Immunology, University of Marburg, Marburg, Germany; ^3^Centre for Biosystems Science and Engineering, Indian Institute of Science, Bangalore, India

**Keywords:** innate immunity, Gram-negative bacteria, macrophage evolution, bacterial niche, phagocytic receptor, antimicrobial protein

## Abstract

Macrophages as immune cells prevent the spreading of pathogens by means of active phagocytosis and killing. We report here the presence of an antimicrobial protein, bactericidal/permeability-increasing protein (BPI) in human macrophages, which actively participates in engulfment and killing of Gram-negative pathogens. Our studies revealed increased expression of BPI in human macrophages during bacterial infection and upon stimulation with various pathogen-associated molecular patterns, *viz*., LPS and flagellin. Furthermore, during the course of an infection, BPI interacted with Gram-negative bacteria, resulting in enhanced phagocytosis and subsequent control of the bacterial replication. However, it was observed that bacteria which can maintain an active replicating niche (*Salmonella* Typhimurium) avoid the interaction with BPI during later stages of infection. On the other hand, *Salmonella* mutants, which cannot maintain a replicating niche, as well as *Shigella flexneri*, which quit the endosomal vesicle, showed interaction with BPI. These results propose an active role of BPI in Gram-negative bacterial clearance by human macrophages.

## Introduction

Innate immune responses refer to the first line of non-specific defense mechanisms that get activated immediately upon encounter with the pathogen. Once a pathogen comes in contact with the host innate immune cells, various sets of genes are upregulated, whose products play an important role in defense mechanisms. These defense mechanisms are classically categorized as O_2_-dependent and O_2_-independent modes of bacterial killing. O_2_-dependent bactericidal activity is mediated by the NADPH phagocyte oxidase and inducible nitric oxide synthase pathways, whereas O_2_-independent bactericidal activity is mediated by antimicrobial peptides and proteins. Bactericidal/permeability-increasing protein (BPI) is a 55-kDa antimicrobial protein with multiple functions including bacterial killing, bacterial opsonization, and LPS neutralization (1). BPI is primarily known to be expressed in human neutrophils and epithelial cells. Previous studies have shown that among innate immune cells, murine BPI is expressed only in dendritic cells and neutrophils but not in macrophages (2). Based on these results, no further studies have been carried out to understand the expression of BPI in macrophages. However, murine macrophages unlike human macrophages are strong producers of nitric oxide and can kill invading pathogens by oxidative stress (3–5). We assumed that in contrast to murine macrophages, human macrophages in compensation for the relatively weak nitric oxide production may employ stronger and more diverse antimicrobial protein production to restrict the spread of invading pathogens. To evaluate this hypothesis, we studied the expression of BPI in human monocytes and macrophages as BPI is the principal O_2_-independent bactericidal agent that acts against Gram-negative bacteria in human neutrophils (6).

In this report, we have analyzed BPI expression in murine and human macrophages under various inflammatory conditions. We investigated the potential role of BPI as an antibacterial agent in human macrophages. Surprisingly, we show that BPI is expressed in human macrophages. In addition to its role as an antibacterial agent, BPI expressed in human macrophages can mediate uptake of Gram-negative bacteria. Gram-negative bacteria which can maintain an active replicating niche in human macrophages avoid the interaction with BPI during later stages of an infection. Together, these results suggest an active role of BPI in Gram-negative bacterial internalization and restricting Gram-negative bacterial replication in human macrophages.

## Results

### BPI Expression in Human Monocytes

**Figure 1:**
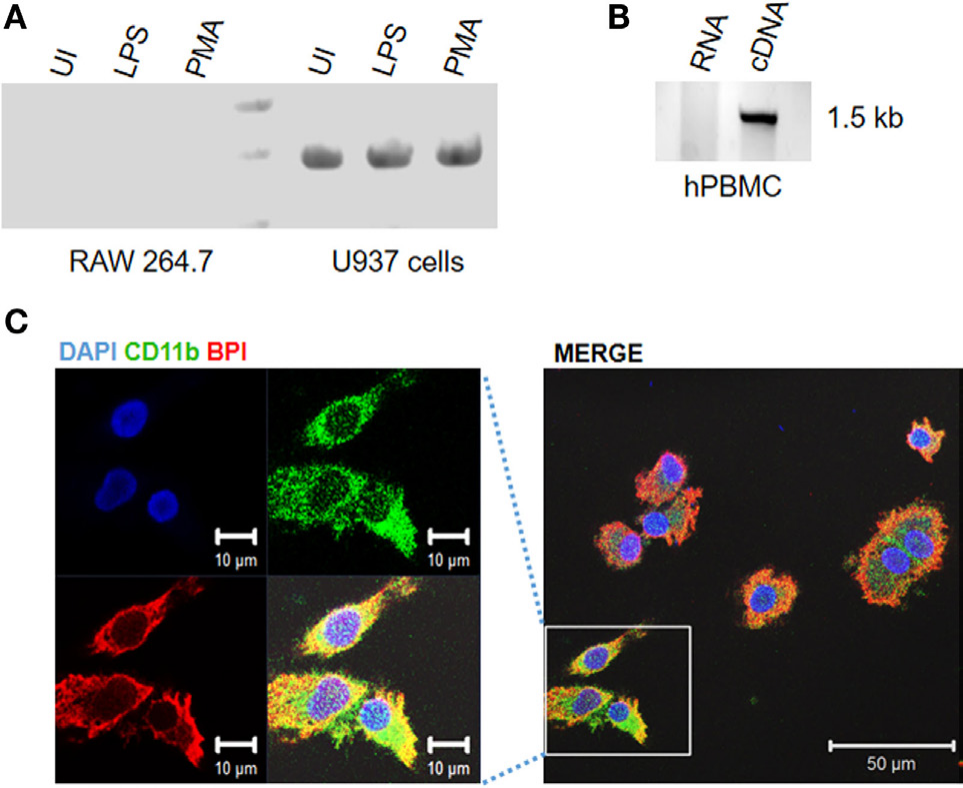
**Bacterial permeability-increasing protein is expressed in human monocyte/macrophages**. Human or mouse monocytes/macrophages were exposed to indicated experimental conditions. Total RNA was extracted from **(A)** human and mouse monocyte and macrophage cell lines, **(B)** human peripheral blood monocytes (hPBMCs) and BPI expression were analyzed by semiquantitative RT-PCR. PCR products were purified and sent for sequencing for further confirmation. **(C)** Human PBMCs were PFA fixed and stained for BPI (red), CD11b (green), and nuclei (blue) and visualized by confocal microscopy.

To understand whether BPI is differentially expressed in human and murine macrophages, BPI expression in murine and human macrophage cell lines were compared in the presence of LPS and PMA (Figure 1A). Surprisingly, BPI mRNA was detected in a human monocyte cell line under resting condition. Furthermore, BPI was found to be expressed in PMA stimulated U937 cells as well, indicating BPI expression in differentiated macrophages (Figure 1A). To validate BPI expression in human PBMCs, BPI-full length product from human PBMCs was amplified and sequenced (Figure 1B). Sequencing results showed >97% sequence identity to BPI-encoding DNA sequence (Figure S1A in Supplementary Material). Additionally, immunostaining with a BPI-specific antibody in human PBMCs revealed predominant localization of BPI toward cell surface (Figure 1C). CD11b staining of human PBMCs further confirmed the presence of BPI in human PBMCs derived macrophages as well as the colocalization of BPI with the surface molecule CD11b (Figure 1C). These results suggest that BPI is expressed in human but not murine macrophages.

### Regulation of BPI Expression in Human Monocytes

**Figure 2:**
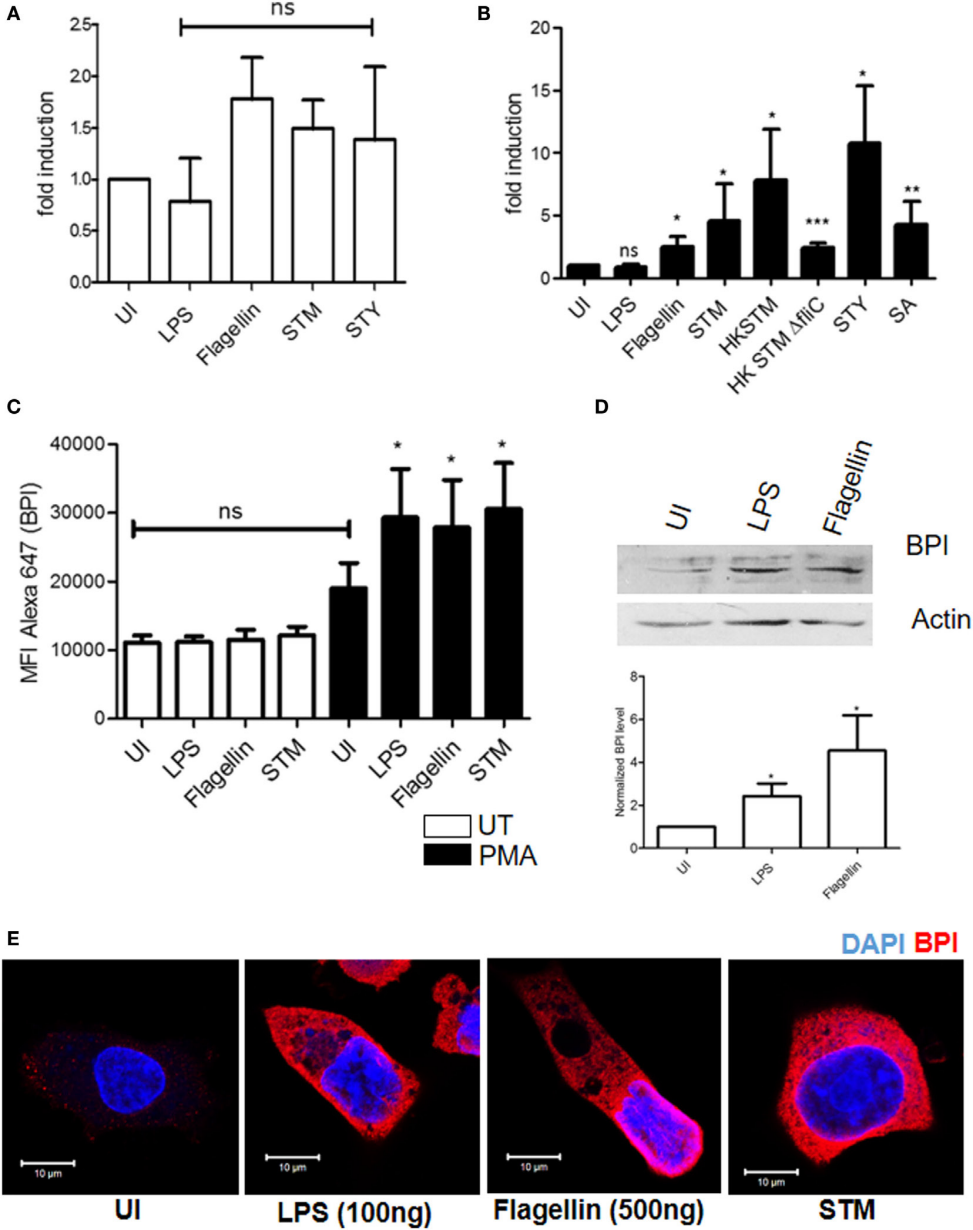
**Regulation of BPI expression in human monocytes/macrophages**. U937 monocytes/macrophages were exposed to indicated inflammatory stimuli/pathogen. **(A,B)** Total RNA was isolated and BPI expression was investigated by real-time PCR [*n* = 5 (SD)]. **(C)** BPI expression was checked by flow cytometry [*n* = 3 (SD)]. **(D)** Total protein was isolated from U937 macrophage, and BPI levels were analyzed by western blot (*n* = 4). Bottom: quantitative evaluation of the relative BPI expression normalized to β-actin intensity. **(E)** Cells were PFA fixed and stained for BPI (red) and nuclei (blue). Cells were imaged by confocal microscopy (*n* = 6). Key: ***p < 0.001, **p < 0.005, *p < 0.05; ns, not significant.

To understand whether BPI expression varies during the course of infection, BPI expression in human monocytes was analyzed under different inflammatory conditions. To this end, U937 cells were infected with different bacteria [*Salmonella* Typhimurium (STM), *Staphylococcus aureus* (SA), and *Salmonella* Typhi (STY)] or were incubated with different PAMPS (LPS and flagellin): afterward, total RNA was isolated and BPI expression was quantified by real-time PCR (Figure 2A). Furthermore, in order to analyze the protein expression of BPI, the cells were fixed and stained with an anti-BPI antibody and investigated by flow cytometry (Figure 2C). These analyses demonstrated that the expression of BPI in human monocytes remained unchanged under all the tested inflammatory conditions.

Bactericidal/permeability-increasing protein is known to be released into inflammatory exudates (7). To understand whether BPI is secreted by human monocytes or macrophages, U937 cells were PMA stimulated or infected with different pathogens (STM and STY). Cell culture supernatant was collected 24 h, post-treatment, and BPI levels were determined by ELISA (Figure S1B in Supplementary Material). There was no detectable level of BPI in cell culture supernatant, indicating that BPI is not secreted by human monocytes or macrophages.

### Regulation of BPI Expression in Differentiated Macrophages

Differentiation of monocytes to macrophages is known to induce an inflammatory and antibacterial response in macrophages (8). To determine whether the increased antibacterial activity of human macrophages is due to increased expression of BPI, U937 cells were differentiated into macrophages by treating monocytes with PMA (50 nM) for 24 h. As expected, differentiated macrophages showed an increased antibacterial activity toward STM compared to U937 monocytes (Figure S2B in Supplementary Material). Interestingly, BPI mRNA expression significantly increased in differentiated macrophages upon treatment with various PAMPs (LPS and flagellin) or infection with various pathogens (STM, STY, and SA) (Figure 2B). BPI expression was increased up to threefold in the presence of flagellin compared to untreated control. In contrast, the BPI expression remained unchanged in the presence of LPS. To evaluate the contribution of flagellin in inducing BPI expression, U937 macrophages were incubated with heat-killed strains of STM [flagellin-deficient *Salmonella* Typhimurium 14028 (STM Δ*fliC*)] and a non-motile Gram-positive pathogen (SA). Heat-killed STM showed increased BPI expression when compared to either untreated controls or HK STM Δ*fliC* and live bacteria. Nevertheless, BPI expression was significantly increased by STM Δ*fliC* and SA compared to untreated control. These results indicate that several PAMPs other than flagellin can also contribute to the BPI expression during infection. Under uninfected conditions, there was no significant increase in BPI mRNA expression in differentiated macrophages compared to U937 monocytes (Figure S2A in Supplementary Material).

In order to evaluate BPI expression at the protein level, U937 macrophages were incubated in the presence of various PAMPs (LPS and flagellin) and bacteria (STM) for 24 h. Thereafter, the cells were fixed and the BPI expression was checked by flow cytometry as well as confocal microscopy (Figures 2C,E). BPI expression was significantly increased in the presence of bacteria as well as PAMPs. LPS induced BPI expression at the protein level, even though there was no significant induction at the RNA level. Western blot analysis showed 2.5-fold increase in BPI expression after LPS treatment and 4.5-fold after flagellin treatment (Figure 2D). These results confirm that BPI is induced in differentiated macrophages during the course of infection.

### BPI Enhances Bactericidal Activity of Human Macrophages

**Figure 3:**
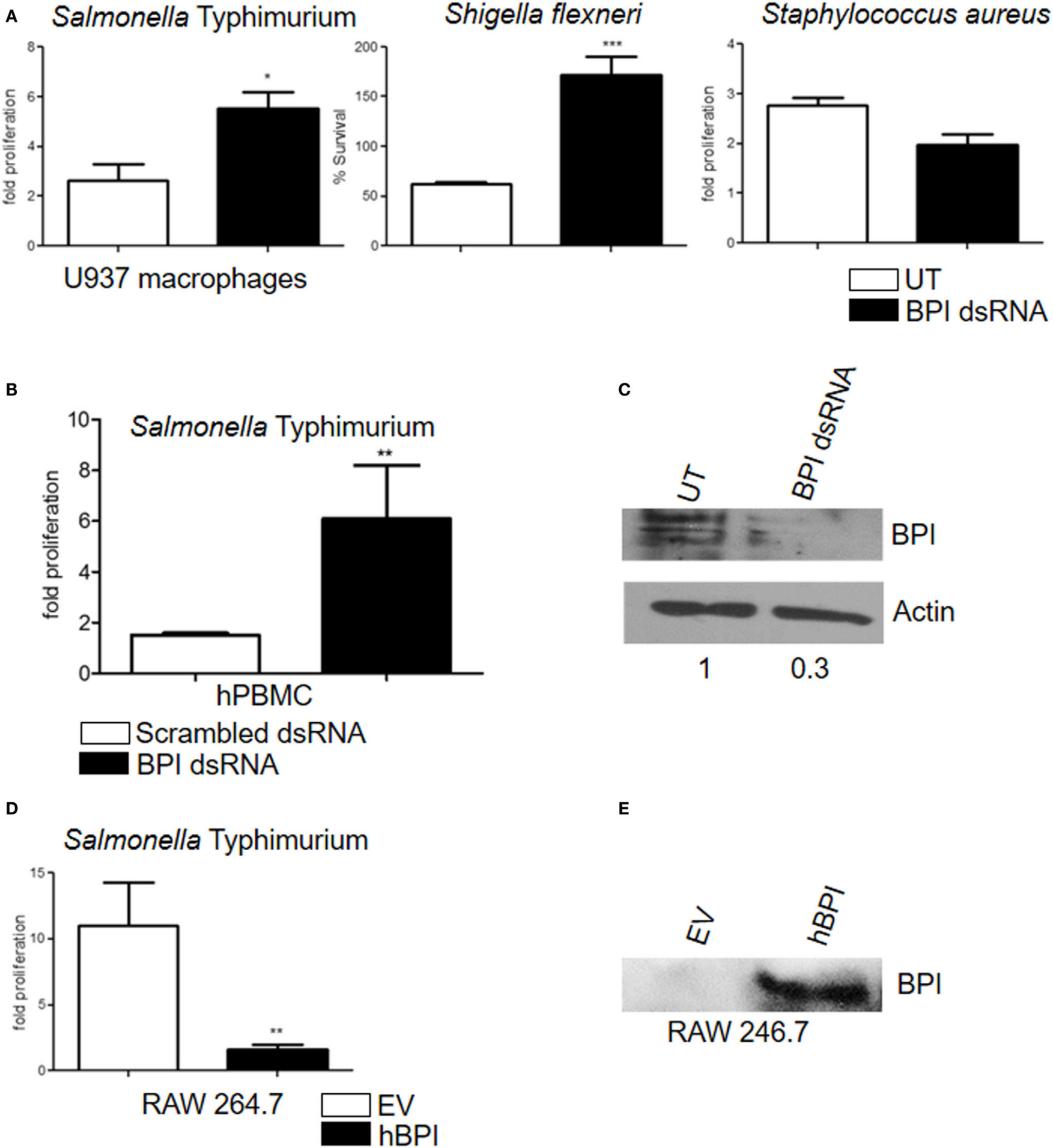
**Human macrophages expressed BPI and kills intracellular bacteria**. **(A)** Fold proliferation and percentage survival of bacteria after knocking down of BPI in U937 macrophages. Statistical significance was calculated with respect to untransfected control [*n* = 5 (SD)]. **(B)** Fold proliferation of STM after knocking down of BPI in human PBMCs-derived macrophages. Statistical significance was calculated with respect to scrambled dsRNA-transfected control [*n* = 6 (SD)]. **(C)** Total protein was isolated from BPI dsRNA-transfected and untransfected control U937 macrophages, and BPI levels were checked by western blot (*n* = 3). **(D)** RAW 264.7 macrophages were transfected with either pcDNA empty vector (pcDNA EV) or pcDNA carrying the expression sequence of human BPI (pcDNA hBPI). Twenty-four hours post-transfection, the cells were infected with STM and bacterial proliferation was quantified [*n* = 4 (SD)]. **(E)** Total protein was isolated from pcDNA EV-transfected and pcDNA hBPI-transfected RAW 264.7 macrophages, and BPI levels were quantified by western blot (*n* = 3). Key: ***p < 0.001, **p < 0.005, *p < 0.05; ns, not significant.

Bactericidal/permeability-increasing protein is known to inhibit the growth of Gram-negative bacteria (9). In order to understand whether BPI expressed in U937 macrophages is functionally active, an antibacterial assay was carried out. U937 macrophages were treated with various PAMPs, which were shown to induce BPI expression (LPS 100 ng and flagellin 500 ng). Twenty-four hours post-treatment, cells were infected with STM 14028 at a multiplicity of infection (MOI) of 10. Bacterial replication was quantified by plating cell lysates 2 and 16 h post-infection. Conditions which induced BPI expression significantly affected bacterial growth in U937 macrophages (Figure S3 in Supplementary Material). To understand the contribution of BPI in inhibiting bacterial growth, bacterial replication was assessed after knocking down BPI in U937 macrophages. To knock down BPI, U937 macrophages were transfected with BPI dsRNA. Twenty-four hours post-transfection, cells were infected with STM 14028 at MOI of 10. The efficacy of BPI knockdown in dsRNA transfected cells was validated by western blot analysis (Figure 3C) and confocal microscopy (Figure S4 in Supplementary Material). Bacterial replication was quantified by plating cell lysates 2 and 16 h post-infection (Figure 3A). Bacterial replication significantly increased in cells where BPI expression was strongly reduced due to dsRNA knockdown compared to untransfected controls. To confirm that BPI expressed in primary human macrophages can inhibit bacterial replication, bacterial replication was assessed in primary human PBMCs after knocking down BPI. Bacterial replication significantly increased in BPI low expressing cells due to dsRNA transfection compared to scrambled dsRNA control (Figure 3B). These data show that BPI contributes significantly in limiting bacterial replication in human macrophages.

Antibacterial activity of BPI is specific toward Gram-negative bacteria due to the specific interaction between BPI and Gram-negative bacterial LPS (6, 10). To validate the specificity of BPI knockdown, Gram-positive bacterial replication was assessed after knocking down BPI in U937 macrophages. U937 macrophages were transfected with BPI dsRNA; 24-h post-transfection, cells were infected with *S. aureus* at an MOI of 10. Bacterial replication was quantified by plating infected cell lysates after 2 and 16 h post-infection (Figure 3A). Replication of *S. aureus* remained unaffected after knocking down BPI in U937 macrophages indicating the specificity of BPI activity to clear Gram-negative pathogens in human macrophages. In order to understand the importance of human BPI in inhibiting bacterial replication, human BPI was amplified from macrophages by PCR, cloned, and expressed in murine macrophages lacking endogenous BPI. Expression of human BPI in RAW 264.7 cells was confirmed by western blotting (Figure 3E). Overexpression of human BPI in murine macrophages increased their antibacterial activity, suggesting that human BPI expressed in human macrophages significantly contributes in bacterial killing (Figure 3D). These results suggest that human BPI actively contributes to the clearance of Gram-negative bacteria in macrophages.

### BPI Mediates Phagocytic Uptake of Gram-Negative Bacteria by Human Macrophages

**Figure 4:**
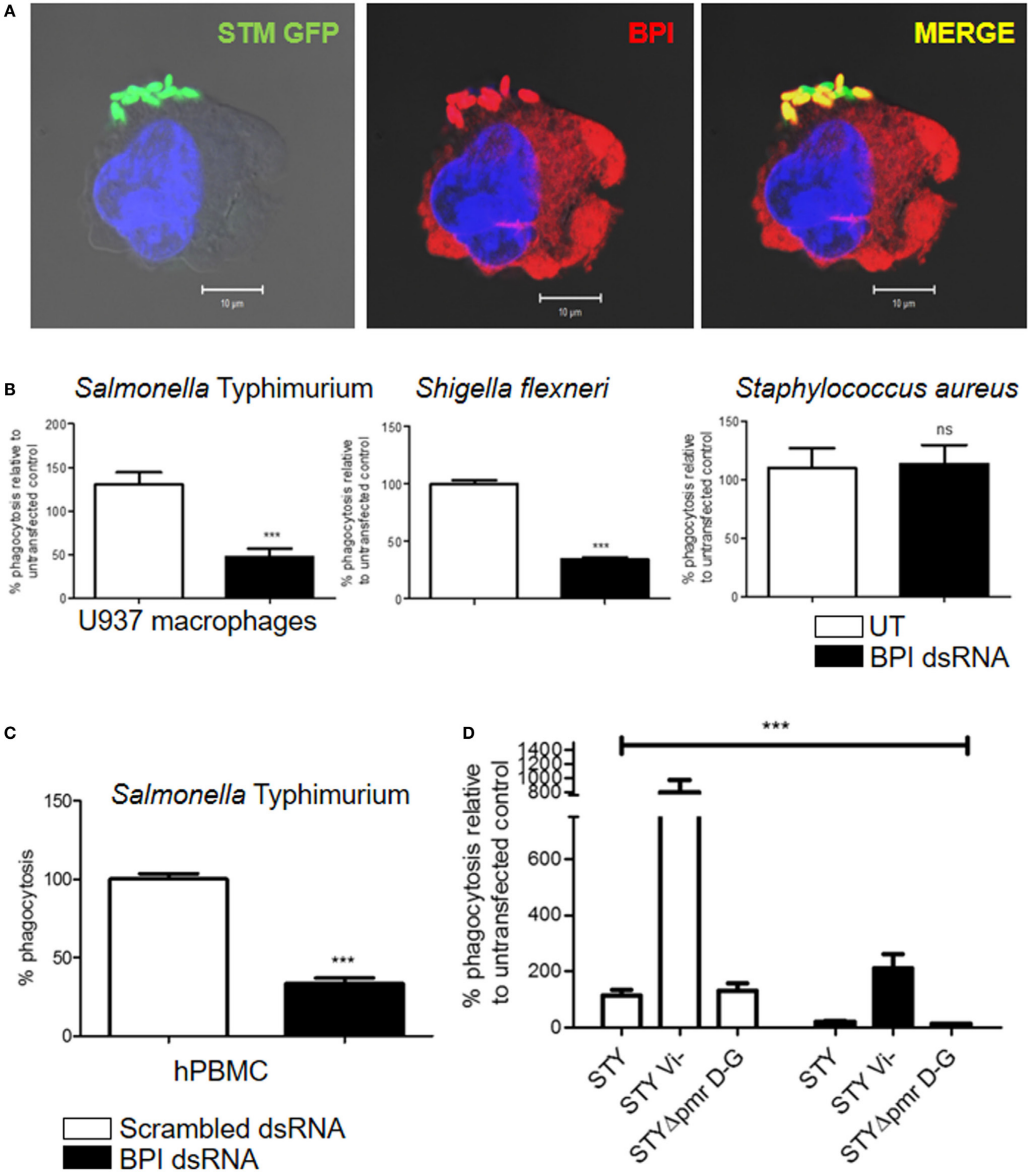
**BPI enhances bacterial uptake in human macrophages**. **(A)** U937 macrophages were infected with STM–GFP (green) at an MOI of 10. Thirty minutes post-infection, cells were fixed with paraformaldehyde and stained for BPI (red) and nuclei (blue) (*n* = 4 experiments). **(B,D)** Percentage phagocytosis of bacteria after knocking down of BPI in U937 macrophages. U937 macrophages were infected with the indicated bacteria at an MOI of 10. Bacterial entry was quantified by plating cell lysates after 30 min post-infection [*n* = 6 (SD)]. For experiments with *Salmonella* Typhi (STY), percentage phagocytosis of BPI dsRNA transfected cells was compared to untransfected control and was normalized to STY (WT). **(C)** Percentage phagocytosis of STM in hPBMC-derived macrophages. Statistical significance was calculated with respect to scrambled dsRNA-transfected control [*n* = 6 (SD)]. Key: ***p < 0.001, **p < 0.005, *p < 0.05; ns, not significant.

In 1997, the role of BPI as an opsonin in human neutrophils was published (11). We observed a significant interaction of surface BPI with Gram-negative bacteria during early time points of infection (Figure 4A). Interestingly, flow cytometry analysis and confocal microscopic analysis of non-permeabilized U937 macrophages showed the presence of BPI on the cell surface. Furthermore, the cell surface-associated BPI interacted clearly with STM (Figure S5 in Supplementary Material). Based on these observations, we hypothesized that BPI expressed on human macrophages might act as a receptor that enhances the phagocytic activity of macrophages toward Gram-negative bacteria. To validate this hypothesis, phagocytosis of Gram-negative bacteria by macrophages was quantified after knocking down BPI in U937 macrophages. U937 macrophages were transfected with BPI dsRNA; 24-h post-transfection, cells were infected with STM 14028 at MOI of 10. Thereafter, phagocytosis of STM 14028 by macrophages was calculated by plating the macrophage cell lysate 30 min post-infection (Figure 4B). We found that the uptake of STM 14028 was significantly decreased upon BPI knockdown in U937 macrophages compared to untransfected control. Interestingly, BPI knockdown did not affect uptake of *S. aureus* (Gram-positive bacteria) by U937 macrophages (Figure 4B). To confirm the role of BPI in Gram-negative bacterial phagocytosis by primary human macrophages, bacterial phagocytosis was assessed in human PBMCs after knocking down BPI. Bacterial uptake was significantly affected upon BPI knockdown in macrophages derived from human PBMCs (Figure 4C). These results suggest that surface expressed BPI contributes significantly in Gram-negative bacterial phagocytosis by human macrophages.

Many pathogenic bacteria are known to possess outer membranous structures that help to evade phagocytosis by macrophages. STY has Vi-polysaccharide that resists opsonophagocytosis mediated by complement receptors (12). The Vi-polysaccharide is also known to inhibit the TLR4-mediated innate immune response (13). BPI and TLR4 recognize lipid A moieties on the surface of bacteria (14). We hypothesized that the presence of Vi capsular polysaccharide might inhibit BPI-mediated phagocytosis of STY. In order to understand the importance of Vi-polysaccharide in BPI-mediated phagocytosis, we checked the percentage phagocytosis of Vi-negative *Salmonella* Typhi (STY Vi^−^) by macrophages in the presence or absence of BPI. BPI was knocked down in U937 macrophages as explained above. U937 macrophages were infected with STY, STY Vi^−^, and STY Δ*pmr*DG. Percentage phagocytosis was calculated by plating the cell lysate 30 min post-infection. Percentage phagocytosis was significantly higher for *Salmonella* devoid of Vi-polysaccharides (STY Vi^−^) compared to STY, but bacterial phagocytosis was significantly decreased irrespective of the presence or the absence of Vi polysaccharide after knocking down BPI in human macrophages (Figure 4D). STY Δ*pmr*DG was used as a negative control in this experiment as the pmr operon is important for structural modifications in LPS but is not important for preventing phagocytosis by macrophages [Figure 4D; (15)]. These results suggest that capsular polysaccharide, although very important to inhibit phagocytosis by macrophages, in general, will not affect the BPI-mediated phagocytosis of Gram-negative bacteria.

### *Salmonella* Typhimurium Evades BPI Interaction during Later Stages of Infection

**Figure 5:**
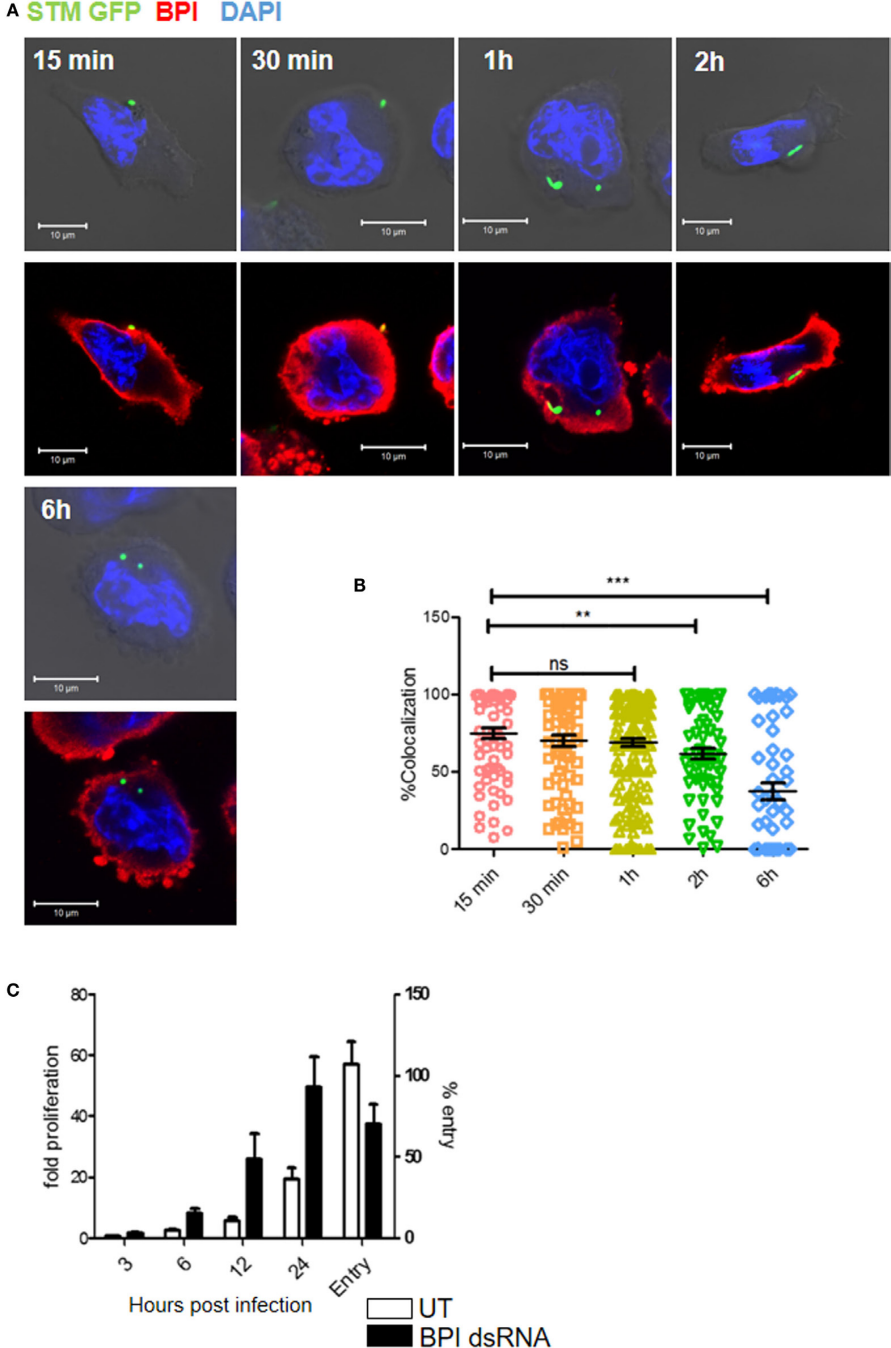
**Time-course analysis of BPI interaction with *Salmonella* Typhimurium in U937 macrophages during the course of infection**. **(A)** U937 macrophages were infected with STM–GFP (green) at an MOI of 50. Cells were fixed with PFA at the indicated time points and stained for BPI (red) and nuclei (blue) (*n* = 3). Panel shows representative bright field and merged images for each time point. **(B)** Quantification of colocalization of BPI with STM–GFP [*n* = 3 (SD)]. **(C)** U937 macrophages were infected with STM–GFP at an MOI of 10. Bacterial entry was quantified by plating the cell lysates after 30 min post-infection. Bacterial replication was quantified by plating the cell lysates at indicated time points and was normalized to the CFU at 1 h post-infection. Fold proliferation and percentage survival were calculated [*n* = 3 (SD)]. Key: ***p < 0.001, **p < 0.005, *p < 0.05; ns, not significant.

We next analyzed the intracellular interaction of BPI with STM by confocal microscopy during the course of infection. Therefore, U937 macrophages were infected with STM14028 at an MOI of 50 and bacterial colocalization with BPI at different time points was analyzed. A region of interest (ROI) was drawn around each bacterium based on GFP signal and % colocalization of a bacterium and BPI at ROI was quantified. STM showed significantly higher colocalization with BPI at early time points of infection (15 min to 1 h). Interestingly, during later time points of infection (2–6 h) by which bacteria maintain a proper niche inside the macrophages, *Salmonella*-containing vesicles (SCVs), a significant lesser colocalization with BPI, could be observed (Figure 5A). The time course of STM replication in macrophages with knocked down BPI showed that STM replication was higher in knocked down conditions within 6 h post-infection compared to untransfected controls. These data are in accordance with previous reports, which suggest that replication of STM takes place starting 6 h post-infection by which the bacteria maintain an actively replicating niche inside the macrophage (16) (Figure 5B). STM replication was significantly higher in BPI KD conditions from 6 to 24 h compared to untransfected control, even though we see a significant decrease in the bacterial entry in BPI KD cells compared to untransfected controls (Figure 5C).

**Figure 6:**
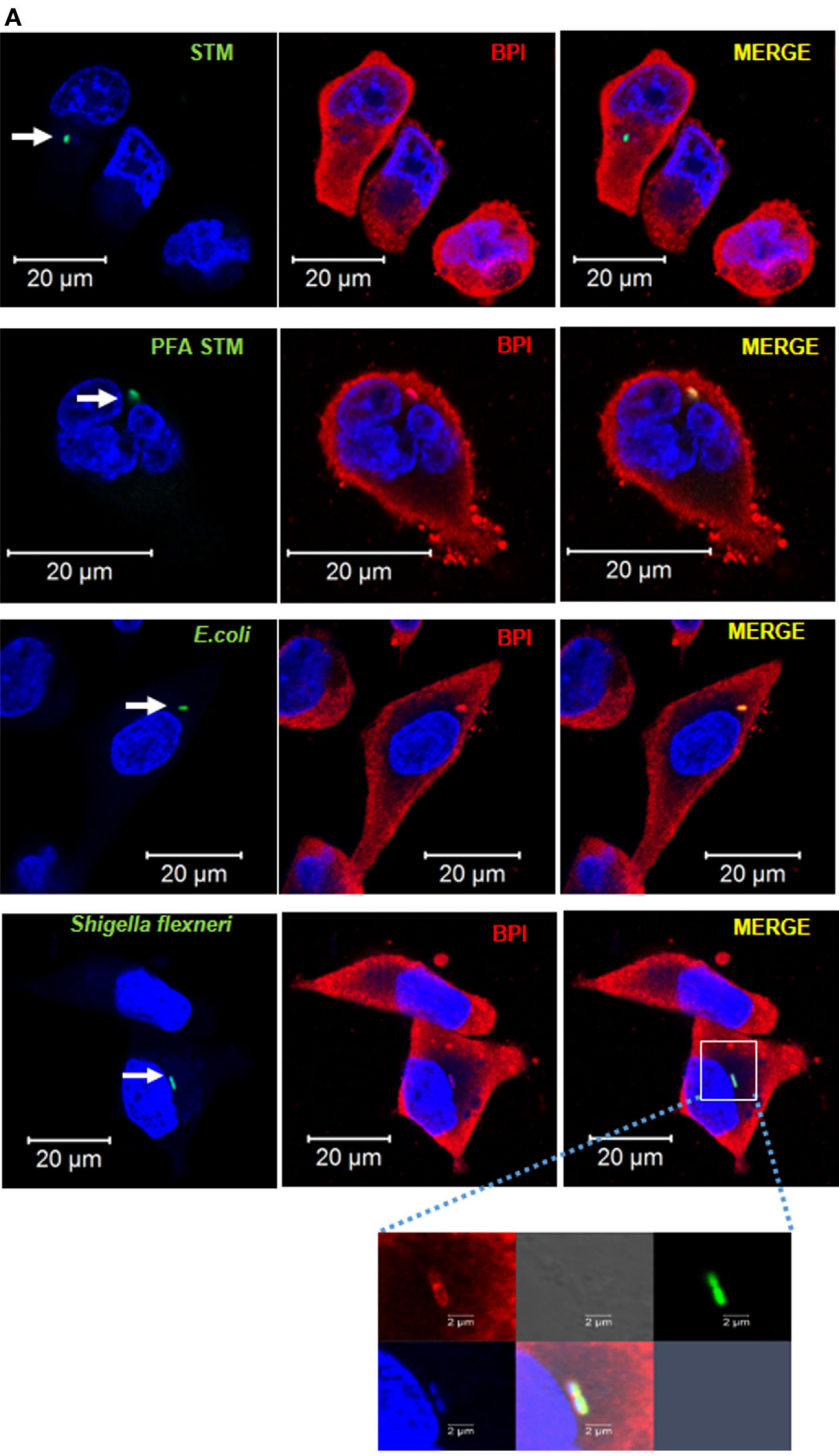

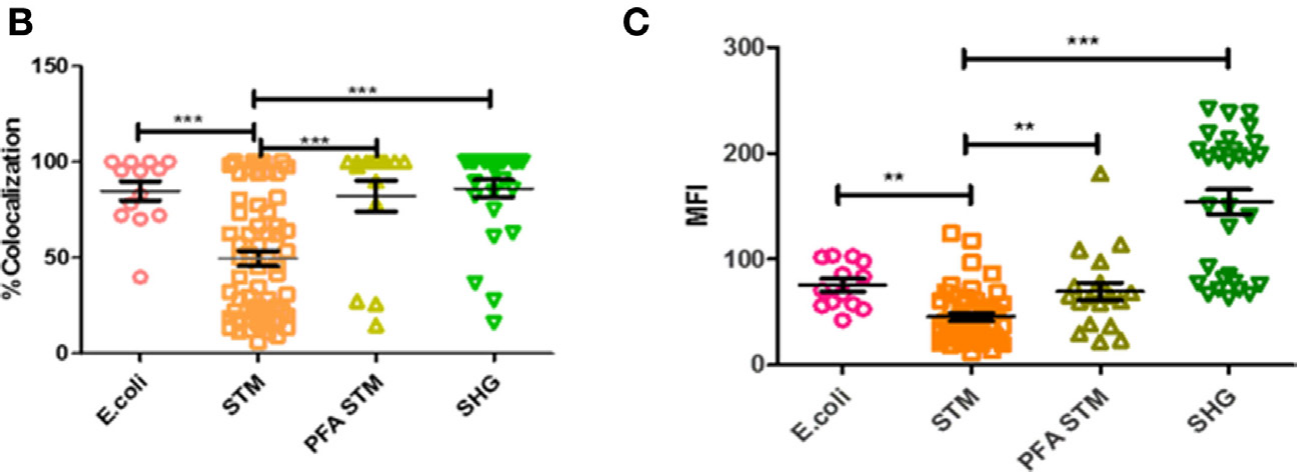
**BPI interaction with Gram-negative bacteria inside macrophages**. **(A)** U937 macrophages were infected with GFP-tagged (green) Gram-negative bacteria [STM14028, PFA fixed STM, *E. coli* DH5α, and *Shigella flexneri (SHG)*] at an MOI of 50. Two hours post-infection, cells were fixed with PFA and stained for BPI (red) and nuclei (blue). White arrows indicate GFP-positive bacteria. The boxed area in the SHG infected set is magnified to view BPI around the bacteria (*n* = 3). **(B)** Quantification of colocalization of BPI with the bacteria. **(C)** Quantification of MFI of BPI at ROI was done by the Zen Blue edition software provided by Zeiss [*n* = 3 (SD)]. Key: ***p < 0.001, **p < 0.005, *p < 0.05.

We next tried to understand the importance of maintaining an actively replicating niche by STM (SCV) to avoid interaction with BPI in human macrophages. U937 macrophages were infected with GFP-tagged bacteria, either replicating STM, paraformaldehyde fixed STM (PFA STM), *Escherichia coli* DH5α (which cannot replicate inside macrophages), or *Shigella flexneri* (SHG; which quits endosomal vesicle). Two hours post-infection, the cells were fixed and the interaction of BPI with the bacteria was analyzed by confocal microscopy (Figure 6A). Recruitment of BPI to the bacteria was analyzed by two methods. First, we checked the interaction of BPI with the bacteria by analyzing the percentage colocalization of BPI and GFP at ROI as explained above (Figure 6B). Second, we analyzed the mean fluorescent intensity (MFI) of BPI at ROI to understand the recruitment of BPI to the bacteria (Figure 6C). There was a significant increase in the recruitment of BPI measured by MFI as well as % colocalization of BPI with PFA STM compared to STM. This may indicate that *Salmonella* actively inhibits the recruitment of BPI to SCV during later stages of infection. Bacteria which cannot replicate inside macrophages (*E. coli*) and bacteria which quit the endosomal vesicle (SHG) showed significantly higher interaction with BPI compared to STM (Figures 6A–C). BPI was found to be localized along the surface of SHG (Figure 6A, inset). In order to evaluate the survival of SHG in human macrophages, we checked the entry as well as the replication of SHG in human macrophages after knocking down BPI as explained above. SHG entry significantly decreased in U937 macrophages upon knockdown of BPI compared to untransfected control (Figure 4B). Interestingly, SHG which usually gets cleared in human macrophages was able to replicate in human macrophages upon knockdown of BPI (Figure 3A). Percentage survival was calculated by normalizing bacterial CFU from 18 h to the CFU count 2 h post-infection. SHG percentage survival was increased from 50 to 150% indicating the importance of BPI in clearing cytosolic bacteria in human macrophages. BPI levels were detected by western blotting after infection with STM, SHG, and *E. coli* in U937 macrophages (Figure S6 in Supplementary Material). There was no significant difference in BPI levels in STM infected cells compared to SHG infected cells. These results indicate that the differential interaction of BPI with STM and SHG is not due to differential expression or degradation of BPI.

### *Salmonella* Typhimurium Maintains an Actively Replicating Niche in Order to Evade BPI Interaction

**Figure 7:**
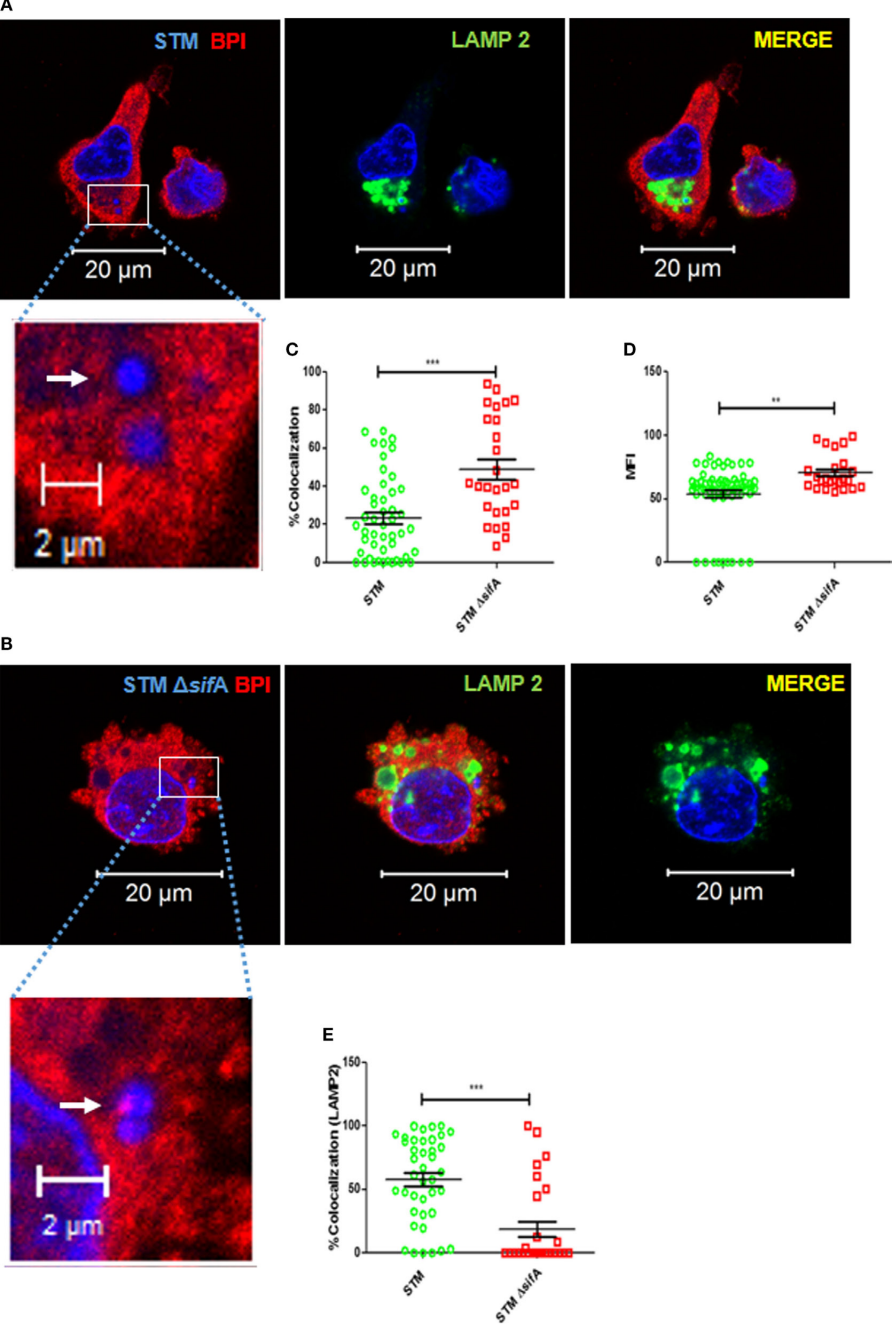

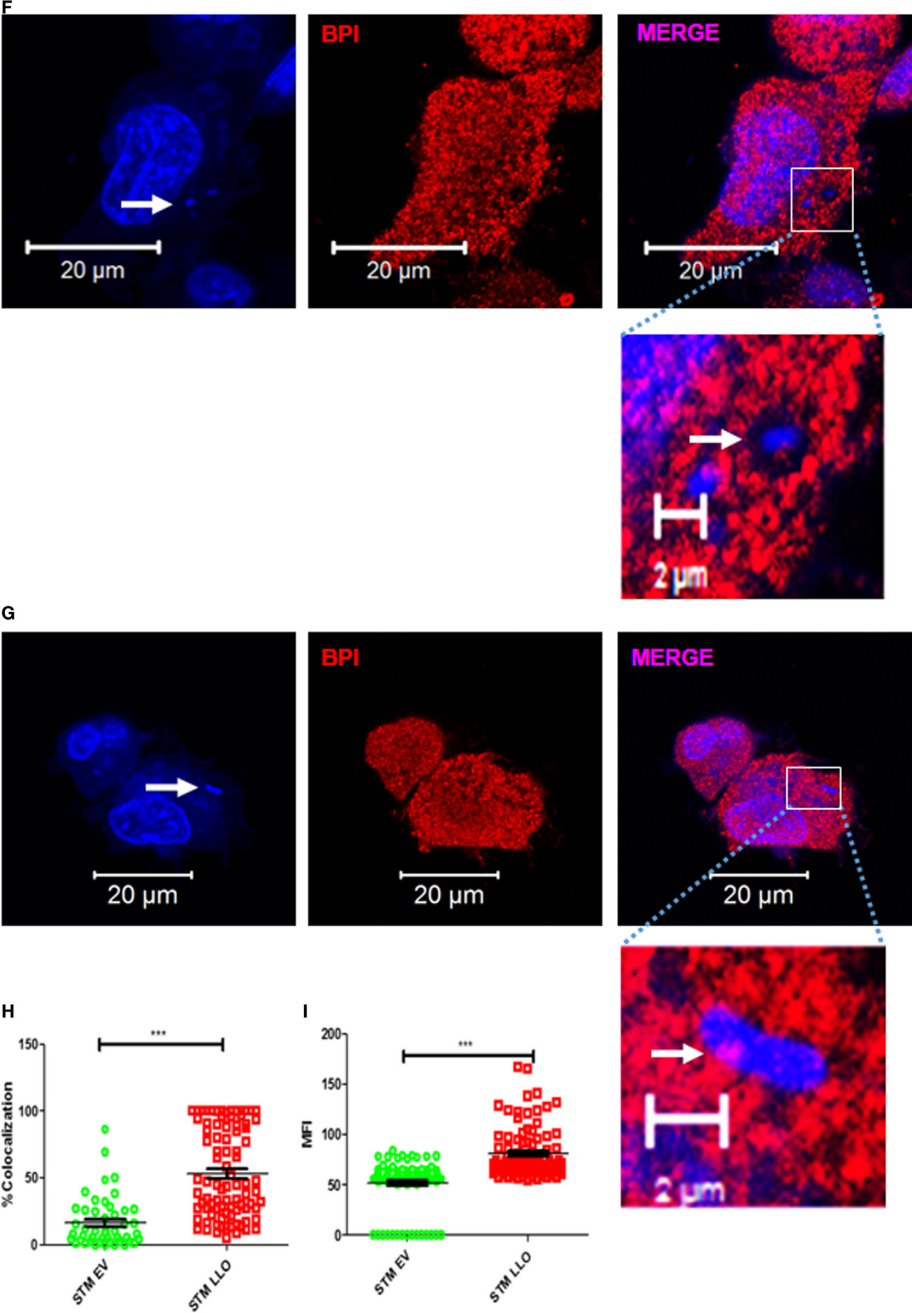
**STM avoids interaction with BPI inside macrophages by maintaining an actively replicating niche (SCV)**. U937 macrophages were infected with **(A)** STM, **(B)** STM Δ*sif*A, **(F)** STM EV (empty vector), or **(G)** STM LLO at an MOI of 50. Two hours post-infection, the cells were fixed with PFA at the indicated time points and stained for BPI (red) and LAMP2 (green). Nuclei and bacteria were labeled with 4′,6-diamidino-2-phenylindole (DAPI) (blue). White arrows indicate DAPI-positive bacteria. The boxed area in each set is magnified to view BPI around the bacteria. **(C,H)** Quantification of colocalization of BPI with bacteria. **(D,I)** Quantification of MFI of BPI at ROI. **(E)** Quantification of colocalization of LAMP2 with bacteria at ROI. All images were quantified by using the Zen Blue edition software provided by Zeiss [*n* = 3 (SD)]. Key: ***p < 0.001, **p < 0.005.

*Salmonella* Typhimurium is known to maintain an actively replicating niche inside the macrophage by modifying the endosomal membrane, thereby preventing their fusion with the late lysosome (SCV) (16). SCV actively modifies its membrane-associated proteins, and these modifications are important for the survival of STM in macrophages. In our present study, we observed that BPI interacts significantly stronger with cytosolic bacteria (SHG) and PFA-fixed STM compared to live STM. These observations led us to hypothesize that SCV might actively avoid the interaction of BPI with the bacteria inside macrophages. To evaluate this hypothesis, we checked the interaction of BPI with STM Δ*sif*A, a *Salmonella* mutant, which cannot maintain an actively replicating niche (SCV) (17). Two hours post-infection, cells were fixed, bacteria and cells were stained with DAPI. An ROI was drawn around each bacterium marked upon DAPI staining (Figures 7A,B). MFI of BPI as well as % colocalization of BPI and bacteria was evaluated at ROI (Figures 7C,D). LAMP 2 was used as a marker to confirm whether STM Δ*sif*A maintains in an intracellular vesicle or not (Figures 7A,B). STM Δ*sif*A mostly remained in the cytoplasm compared to STM wild type, which was in LAMP2-positive compartments as analyzed by LAMP2 colocalization (Figure 7E). STM Δ*sif*A showed an increased colocalization with BPI compared to STM wild type, indicating the importance of the vacuolar life of *Salmonella* in maintaining a replicative niche devoid of BPI inside macrophages. STM Δ*sif*A showed an increased recruitment of BPI around the bacteria measured by evaluating MFI as well as the percentage colocalization at ROI (Figures 7C,D).

To confirm these results, we checked bacterial interaction with BPI under conditions which make bacteria quit the vesicle. To attain this, we expressed listeriolysin (LLO) in STM. LLO is known to make pores into vesicular membranes, which lead to the rupturing of the membrane (18). LLO expression was induced in STM LLO using IPTG. U937 macrophages were infected with STM EV (empty vector) or STM LLO. Two hours post-infection, cells were fixed and bacteria and cells were stained with DAPI (Figures 7F,G). An ROI was drawn around each bacteria marked upon DAPI staining. MFI of BPI, as well as % colocalization of BPI and bacteria, was evaluated at ROI. BPI and STM LLO colocalized significantly higher compared to STM EV as seen by increased % colocalization in STM LLO in comparison to STM EV (Figure 7H). Recruitment of BPI as measured by checking the MFI at ROI was also higher in STM LLO compared to STM EV (Figure 7I). Under these conditions, STM LLO showed a twofold decrease in bacterial proliferation compared to STMEV control (Figure S7 in Supplementary Material). These results suggest that STM avoids interaction with BPI by maintaining an actively replicating niche (SCV) inside macrophages.

## Discussion

Bactericidal/permeability-increasing protein is known to be expressed in human neutrophils, epithelial cells, eosinophils, and the genital tract (1). Previous studies by Buurman et al. (19) suggested the expression of BPI in human monocytes, but the experimental evidence clearly did not prove whether BPI is expressed in human monocytes or whether BPI is adsorbed on to the monocyte surface. In this current study, we clearly demonstrate the expression of BPI in human macrophages both at RNA and protein levels. Interestingly and in sharp contrast, under similar conditions, we could not detect BPI expression in murine macrophages. The reactive nitrogen species (RNS)-mediated antibacterial activity of human macrophages is still a controversy (20). On the one hand, there are reports which suggest that unlike murine macrophages, human macrophages cannot produce RNS (5). On the other hand, RNS could be detected in human macrophages isolated from PBMCs of infected patients. Regardless these reports, in summary, suggest that NO production by human macrophages is either extremely low or requires complex signaling mechanisms for induction (4, 5). Our results on the expression of BPI only in human macrophages suggest that human macrophages during the course of evolution might have obtained a strong bias toward O_2_-independent mechanisms to kill pathogens as exemplified by BPI. This might give an advantage to the host to avoid free radical-mediated damage to the host cells associated with reactive nitrogen species produced during infection and in circumstances when oxygen tension is low.

Differentiation of monocytes to macrophages is known to increase the antibacterial activity of human macrophages (8). The precise mechanistic aspects of increased antibacterial activity of differentiated macrophages are not known. In our current study, we show that BPI expression is increased in differentiated macrophages upon bacterial infection, whereas in undifferentiated monocytes, there was no significant increase in BPI expression. Interestingly, knocking down of BPI in macrophages led to the proliferation of three different strains of Gram-negative bacteria tested, but did not affect proliferation of *S. aureus*. The signaling pathways that lead to BPI induction in human macrophages are not clear. In our study, we saw that all the PAMPs tested can induce BPI expression. These results show that BPI might be induced as a general antimicrobial protein during the infection by any pathogen in macrophages. Interestingly, LPS increase BPI protein level without changing the BPI mRNA level. This might be due to an increase in the stability of BPI mRNA mediated by MyD88 signaling pathway (21).

Previous results by Weiss and group demonstrated that BPI secreted by neutrophils can act as an opsonin and can induce opsonophagocytosis by macrophages (11). We could not detect BPI secreted by human macrophages upon infection. Interestingly, most of the BPI expressed in human macrophages is present on the cell surface. During the early stages of infection, BPI significantly interacted with Gram-negative bacteria (Figure 5B). Knocking down of BPI in human macrophages led to a reduced entry of Gram-negative bacteria. These results may suggest that apart from acting as an opsonin, BPI expressed on the cell surface can itself act as a receptor for binding of Gram-negative bacteria. Whether this binding can itself induce phagocytosis or whether BPI helps the bacteria to adhere to macrophages and other phagocytic receptors distinct from the interaction with BPI can induce phagocytosis of bacteria is not clear. Recently, a report by Casanova et al. showed that GPCR brain-specific angiogenesis inhibitor 1 (BAI 1) can act as a receptor for the uptake of Gram-negative bacteria and can also induce bacterial killing by indirect means (22). BPI may also act as a phagocytic receptor, but unlike BAI 1, BPI itself can act as a direct bactericidal agent. Irrespective of the presence or absence of capsular material, BPI can induce phagocytosis of Gram-negative bacteria as seen by phagocytosis of Vi-positive *S. Typhi*. How antimicrobial peptides and proteins can interact with LPS of capsulated bacteria is still not clear. We believe that the extensions of LPS outside capsular polysaccharide might act as a docking site for BPI (11). Whether BPI can interact with the capsular polysaccharide itself is something that should be explored in the future. Since BPI-mediated phagocytosis did not need any opsonin, it will be interesting to check the level of BPI in macrophages which are present in opsonin poor environment (e.g., alveolar macrophages). BPI significantly interacted with STM during early time points of the infection. During later time points of the infection, STM avoids BPI interaction during later time points and thereby maintains an actively replicating niche inside macrophages. Bacteria which cannot maintain an actively replicating niche (SHG, *E. coli*) will be cleared easily by the macrophages. All these bacteria tested showed significant colocalization with BPI during later time points of infection as well. Interestingly, SHG which cannot multiply in human macrophages started multiplying in BPI knockdown macrophages. Confocal analysis showed the presence of BPI around SHG during later time points of the infection indicating the importance of BPI in controlling *Shigella*. Interestingly, once the actively dividing bacteria (STM) leaves the replicating niche (STM LLO, STM Δ*sif*A), they will interact with BPI. These results demonstrate an active role of BPI in eliminating Gram-negative bacterial pathogens inside macrophages. Whether BPI can cross talk with other signaling pathways and can induce additional bactericidal activity is not entirely clear. It will be interesting to analyze the contribution of macrophage-derived BPI in preventing various infectious diseases including parasitic and bacterial infections. Polymorphisms in BPI are associated with different inflammatory diseases, including Crohn’s disease (CD) (23–25). Macrophages derived from patients with CD show impaired bacterial clearance (26, 27). Whether this impaired clearance of bacteria is due to polymorphisms of BPI in macrophages derived from CD patients is yet to be understood.

## Methods

### Cell Culture

The human monocyte cell line U937 (NCCS, Pune) and murine macrophage cell line RAW 264.7 (kind gift from Prof. Anjali Karandae, IISc) were maintained in RPMI (Sigma-Aldrich) containing 10% FBS (fetal bovine serum, Gibco). For induction of macrophage differentiation, cells were seeded and stimulated with 50-nM PMA (phorbol 12-myristate 13-acetate, Sigma-Aldrich) for 24 h. After PMA induction, non-attached cells were removed by gentle aspiration and attached cells were washed three times with RPMI containing 10% FBS.

### Knockdown of BPI in Human Macrophages

In order to knock down BPI in human macrophages, BPI dsRNA was designed against three regions within the gene: (a) GGAGCTGAAGAGGATCAAGATTCCTGACTACTCAGACAGCTTTAAGATCAAGCATCTTGGGAAGGGGCATTATAGCTTCTACAGCATGGACATCCGTGAATTCCAGCTTCCCAGTTCCCAGATAAGCATGGTGCCCAATGTGGGCCTTAAGTTCTCCATCAGCAACGCCAATATCAAGATC; (b) TGTCCACGTGC ACATCTCAAAGAG CAAAGTCGGGTGGCTGAT CCAACTCTTCCACAAAAAAATTGAGTCTGCGCTTCGAAAC AAGATGAACAGCCAGGTCTGCGAGAAAGTGACCAATTCTGTATCCTCCAAGCTGCAACCTTATTTCCAGACTCTGC; and (c) GGGTCTTGAAGATGACCCTTAGAGATGACATGATTCCAAAGGAGTCCAAATTTCGACTGACA ACCAAGTTCTTTGGAACCTTCCTACCTGAGGTGGCCAAGAAGTTTCCCAACATGAAGATACAGATCCATGTCTCAGCCTCCACC. All dsRNA were obtained from chromous biotech. Transfection was done using oligofectamine as recommended by the manufacturer (Invitrogen, Life Technologies). Transfection was done for 24 h.

### Cloning and Expression of Human BPI in Murine Macrophages

The complete BPI coding sequence was amplified from cDNA derived from human monocytes. PCR product was gel eluted and was cloned in pcDNA 3.1 expression vector (kind gift from Dr. G. Subbha Rao, IISc). The pcDNA 3.1 hBPI was transiently transfected to RAW macrophages using PEI transfection reagent (Sigma-Aldrich). Twenty-four hours post-transfection, cells were harvested and BPI expression was quantified by western blotting. Transiently transfected cells were used for infection assay. pcDNA 3.1 was used as empty vector control in all the experiments. The sequence for cloning primers is as follows: hBPI forward primer, 5′AAGGATCCATGAGAGAGAACATGGCC3′, and hBPI reverse primer, 5′GGCAAGCTTTCATTTATAGACAACGTC3′. The restriction sites within the primers are underlined.

### Bacterial Strains and Growth Conditions

*Salmonella* Typhimurium (*Salmonella* enerica serovar Typhimurium ATCC14028s), STM Δ*fliC* (flagellin-deficient STM 14028), STY (STY ATCC CT18), SHG (SHG clinical isolate 1), SA (SA ATCC 25923), and *E. coli* (*E. coli* DH5α ATCC) were grown in Luria-Bertani medium at 37°C. STM Δ*sif*A and STM Δ*fliC* was a gift from Michael Hensel, Universität Osnabrück, Germany. For immunostaining experiment STM, SHG and *E. coli* were transformed with the pFPV25.1 plasmid containing the GFPmut3 gene (Addgene). In order to express listeriolysin O in STM, STM 14028 was transformed with pPROEX HT-b LLO (STM LLO). STM 14028 transformed with pPROEX HT-b (STM EV) was used as empty vector control. pPROEX HT-b LLO was a kind gift from Prof Sandhya S. Visweswariah, IISC. All the transformants were maintained in Luria-Bertani medium containing 100 μg/ml of ampicillin. For induction of LLO expression in STM LLO, log phase cells were treated with 500-nM IPTG (Sigma-Aldrich) for 6 h. STY Vi^−^ (STY lacking Vi polysaccharide) was a kind gift from Prof Ayub Qadri, NII.

### Immunofluorescence Microscopy

U937 cells were seeded on glass coverslips overnight before infection or treatment. After treatment, cells were washed with PBS and fixed with 3.5% paraformaldehyde for 15 min. Cells were permeabilized using 1% saponin (Sigma-Aldrich) dissolved in PBS with 3% BSA. Immunostaining was done using anti-BPI antibody (Sigma-Aldrich) followed by anti-rabbit Alexa 647 antibody (DSHB, University of Iowa). To visualize macrophage population in human PBMC cultures, cells were stained with the anti-CD11b antibody (DSHB, University of Iowa) followed by anti-mouse Alexa 488 antibody (DSHB, University of Iowa). To label lysosomes, anti-LAMP2 antibody (DSHB, University of Iowa) was used followed by anti-mouse Alexa 488 antibody (DSHB, University of Iowa). Cells were counterstained using DAPI (Sigma-Aldrich) to label the nucleus. To visualize bacteria, either pFPV25.1 GFPmut3-transformed bacteria were used or bacteria were visualized using DAPI staining. Image acquisition was done with a Zeiss confocal microscope (LSM Meta 710). Quantitation of images was done as explained by Billings et al. (22). Briefly, for quantification of BPI recruitment and interaction with bacteria, an ROI was drawn around each bacterium based upon GFP signal (STM–GFP, SHG–GFP, and *E. coli* GFP) or DAPI staining (STM Δ*sif*A, STM LLO, and STM EV). Images were analyzed using Zen Blue edition software provided by Zeiss. The colocalization coefficient values at ROI were obtained using Zen Blue edition software and were multiplied by 100 to get the percent colocalization and plotted. The MFI of BPI at ROI was calculated Zen software and plotted.

### Bacterial Phagocytic Uptake and Proliferation Assay

Bacteria were grown in Luria-Bertani medium, and overnight culture was used to infect U937 cells at a ratio of 10 bacteria per cell (MOI 10). Extracellular bacteria were removed 30 min post-infection, and cells were maintained in 100 μg/ml gentamycin for 1 h to kill any extracellular bacteria. Infected cells were maintained in DMEM containing 10 μg/ml of gentamycin. Phagocytosis of bacteria by macrophages was calculated by plating the macrophage cell lysates 30 min post-infection. For calculating the percentage phagocytosis, CFU was normalized with respect to untransfected control. The obtained value was multiplied by 100 to get percentage phagocytosis.

%Phagocytosis=(CFU of Test/CFU of Untransfected control)×100

Bacterial replication inside macrophages was quantified by plating the cell lysates 2- and 18-h post-infection. Fold proliferation was calculated by normalizing the bacterial CFU at 18 h with respect to 2 h. For experiments using SHG, the bacterial survival was calculated instead of fold replication because SHG cannot proliferate inside macrophages.

Fold proliferation=CFU of bacteria at 18 h/CFU of bacteria at 2 h

%Survival(SHG)=(CFU of SHG at 2 h/CFU of SHG at 18 h)×100

### Infection and Stimulation of Human Macrophages

For stimulation with various inflammatory mediators, PMA-treated U937 cells were incubated with STM LPS 100 ng/mL (Sigma-Aldrich) and STM flagellin 500 ng/mL in DMEM containing 10% FBS for the indicated time periods. Flagellin was isolated from STM as previously described in details (28). U937 monocyte cell line was used as a control throughout the experiment.

### Human PBMC Isolation

This study was approved by Institutional Biosafety guidelines (IBSC) at Indian Institute of Science, Bangalore, India (Ref No: IBSC/IISC/DC/04/2015), and written informed consent was obtained from all participants before participation. All the procedures were carried out by trained medical technician. Human PBMCs were isolated from healthy individuals using Himedia LSM as per instructors manual. Briefly, blood collected from healthy individuals were overlaid on LSM and separated into different layers using a low-speed centrifugation. The cell layer containing human PBMCs was collected after centrifugation and mixed with DMEM without serum. Cells were seeded into a six-well plate, and unattached cells were gently aspirated. Attached cells were washed three times with DMEM containing 10% FBS and maintained in the same for 24 h.

### PCR and Real-time Analysis of BPI mRNA Expression

Total RNA was extracted from 1 × 10^6^ cells using TRIzol reagent (Invitrogen) as per the manufacturer’s protocol. After DNase treatment, 2 μg of RNA was used for cDNA synthesis using tetra-reverse transcriptase (Bioline). qRT-PCR was performed using the Kapa SYBR Green RT-PCR kit (Kapa Biosystems) as per the manufacturer’s protocol in an Applied Biosystems^®^ ViiA™ 7 Real-Time PCR instrument. The following primers were used for detecting BPI level by real-time PCR: hBPI forward primer, 5′ATGAACAGCCAGGTCT 3′, and hBPI reverse primer, 5′GGTCATTACTGGCAG 3′. Expression was normalized to the housekeeping gene beta-actin. Following primers were used for detecting actin level: actin forward primer, 5′GGTGGCTTTTAGGATGGCAAG3′, and actin reverse primer, 5′ACTGGAACGGTGAAGGTGACAG3′. Expression levels were calculated using the 2^−δδCt^ method.

### Western Blotting and FACS to Quantify Protein Levels

For quantifying BPI expression by western blot, 10^6^ cells were grown on six-well plates and exposed to various conditions as mentioned. Cell lysates were prepared and proteins were resolved by 10% SDS-PAGE and transferred to PVDF membrane. The blots were incubated with an anti-BPI antibody (Sigma-Aldrich) followed by anti-rabbit HRP (DSHB, University of Iowa). Immunoblots were visualized by ECL reagent. Densitometric quantification of blots was done using the Multi Gauge software (FUJIFILM).

For quantification of protein expression by flow cytometry, cells were fixed using 3.5% paraformaldehyde for 15 min. Cells were permeabilized using 0.1% saponin dissolved in PBS with 3% BSA. Immunostaining was done using anti-BPI antibody (Sigma-Aldrich) followed by anti-rabbit Alexa 647 antibody (DSHB, University of Iowa). Cells were subjected to flow cytometric analysis (BD FACSCalibur™). Data were analyzed using BD FACSDIVA™ software.

### Statistical Analysis

The data were subjected to statistical analysis by applying Student’s *t*-test by using Graph Pad prism 4 software.

## Author Contributions

AB and DC conceived the study; AB performed the experiments; and AB, DC, and MS analyzed the data and wrote the manuscript.

## Conflict of Interest Statement

The authors declare that the research was conducted in the absence of any commercial or financial relationships that could be construed as a potential conflict of interest.
