# Supplementary material for: Of Men Not Mice: Bactericidal/Permeability-Increasing Protein Expressed in Human Macrophages Acts as a Phagocytic Receptor and Modulates Entry and Replication of Gram-Negative Bacteria
Source: Front Immunol. 2016 Oct 24;7:455. doi: 10.3389/fimmu.2016.00455 (PMC5075746; doi:10.3389/fimmu.2016.00455)
Supplement: Supplementary file 1 [file Presentation_1.PPTX]

## Slide 1
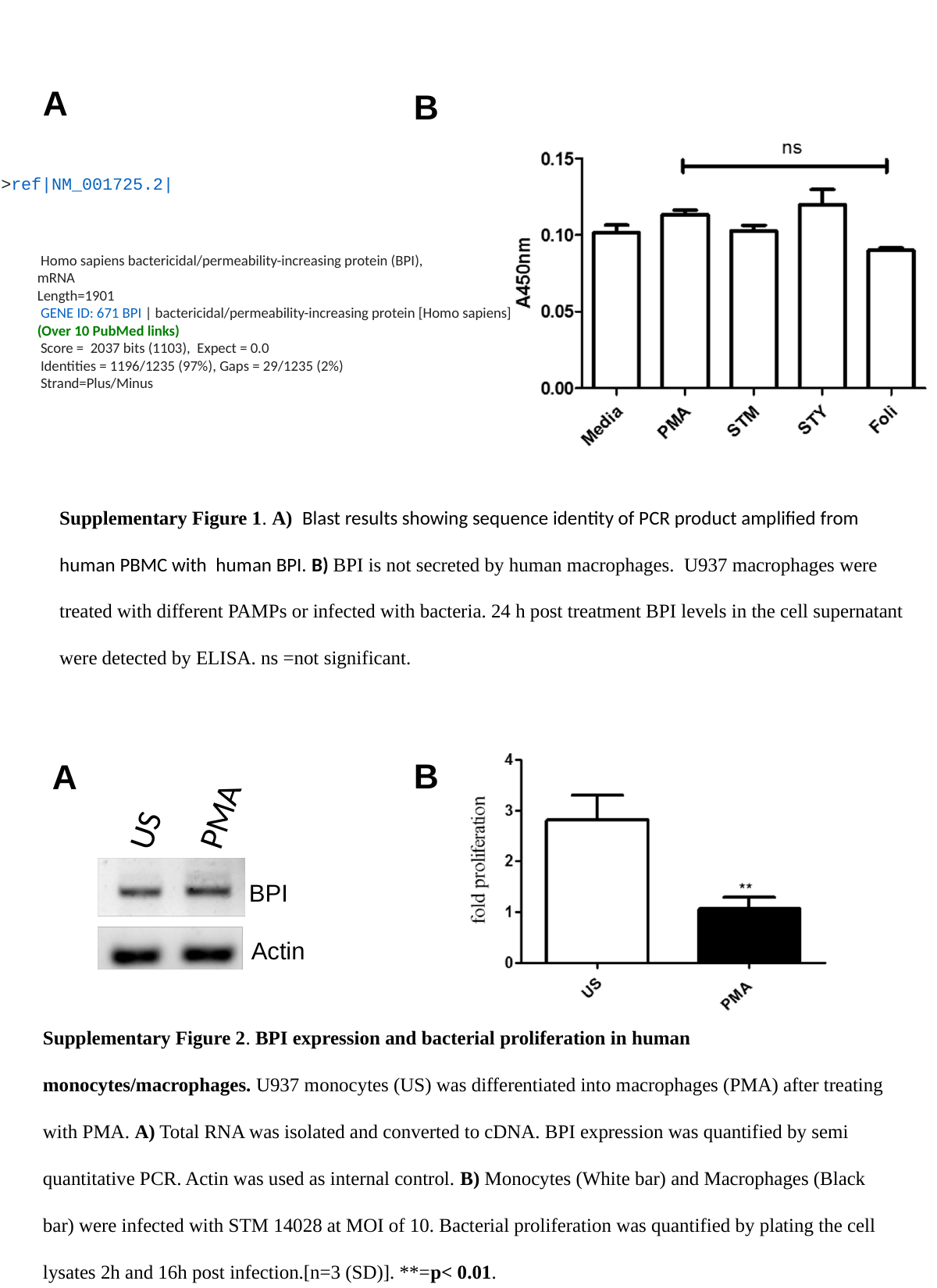

A
B
>ref|NM_001725.2|
 Homo sapiens bactericidal/permeability-increasing protein (BPI),
mRNA
Length=1901
 GENE ID: 671 BPI | bactericidal/permeability-increasing protein [Homo sapiens]
(Over 10 PubMed links)
 Score = 2037 bits (1103), Expect = 0.0
 Identities = 1196/1235 (97%), Gaps = 29/1235 (2%)
 Strand=Plus/Minus
Supplementary Figure 1. A) Blast results showing sequence identity of PCR product amplified from human PBMC with human BPI. B) BPI is not secreted by human macrophages. U937 macrophages were treated with different PAMPs or infected with bacteria. 24 h post treatment BPI levels in the cell supernatant were detected by ELISA. ns =not significant.
PMA
US
BPI
Actin
B
A
Supplementary Figure 2. BPI expression and bacterial proliferation in human monocytes/macrophages. U937 monocytes (US) was differentiated into macrophages (PMA) after treating with PMA. A) Total RNA was isolated and converted to cDNA. BPI expression was quantified by semi quantitative PCR. Actin was used as internal control. B) Monocytes (White bar) and Macrophages (Black bar) were infected with STM 14028 at MOI of 10. Bacterial proliferation was quantified by plating the cell lysates 2h and 16h post infection.[n=3 (SD)]. **=p< 0.01.

## Slide 2
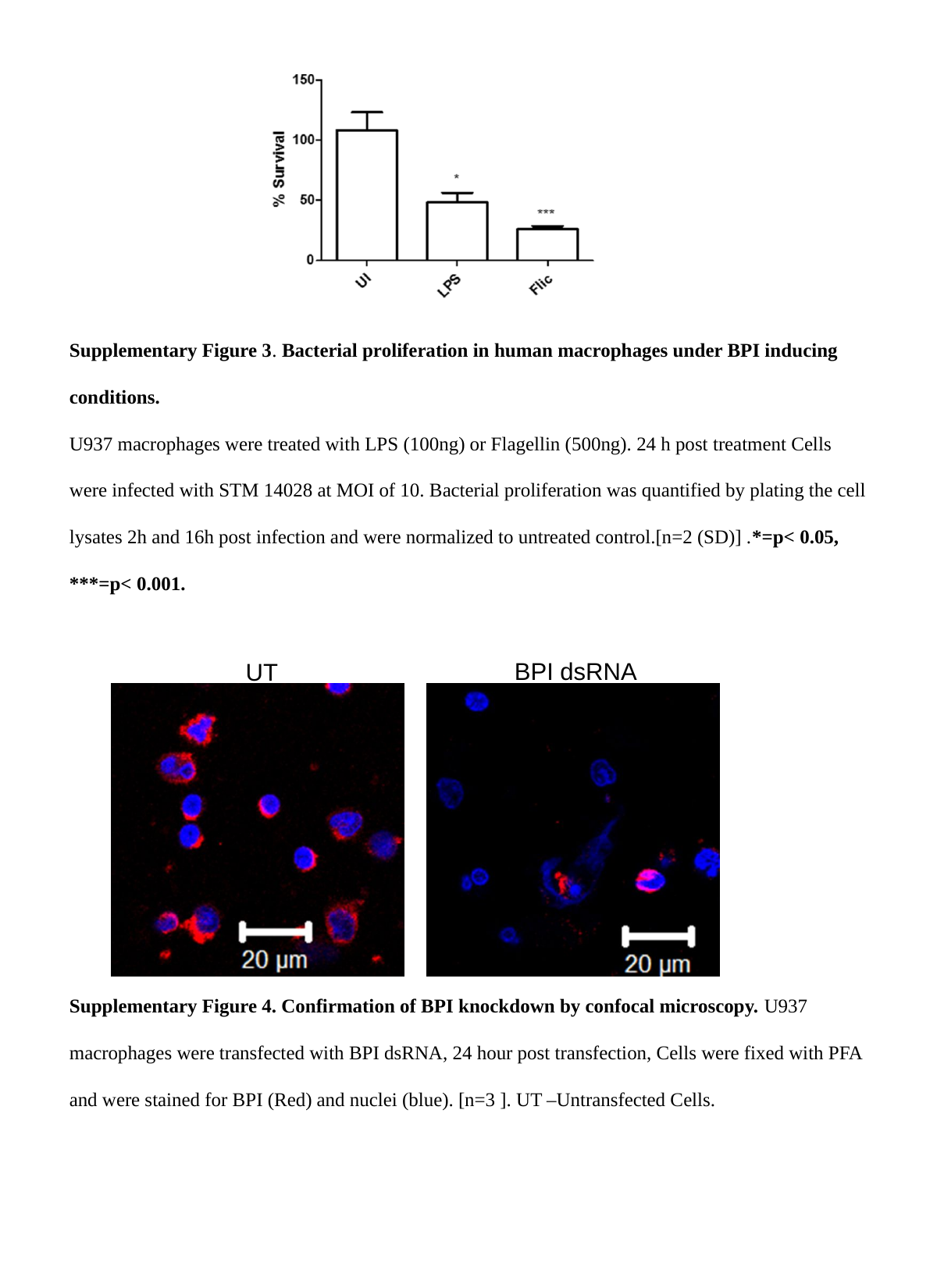

Supplementary Figure 3. Bacterial proliferation in human macrophages under BPI inducing conditions.
U937 macrophages were treated with LPS (100ng) or Flagellin (500ng). 24 h post treatment Cells were infected with STM 14028 at MOI of 10. Bacterial proliferation was quantified by plating the cell lysates 2h and 16h post infection and were normalized to untreated control.[n=2 (SD)] .*=p< 0.05, ***=p< 0.001.
BPI dsRNA
UT
Supplementary Figure 4. Confirmation of BPI knockdown by confocal microscopy. U937 macrophages were transfected with BPI dsRNA, 24 hour post transfection, Cells were fixed with PFA and were stained for BPI (Red) and nuclei (blue). [n=3 ]. UT –Untransfected Cells.

## Slide 3
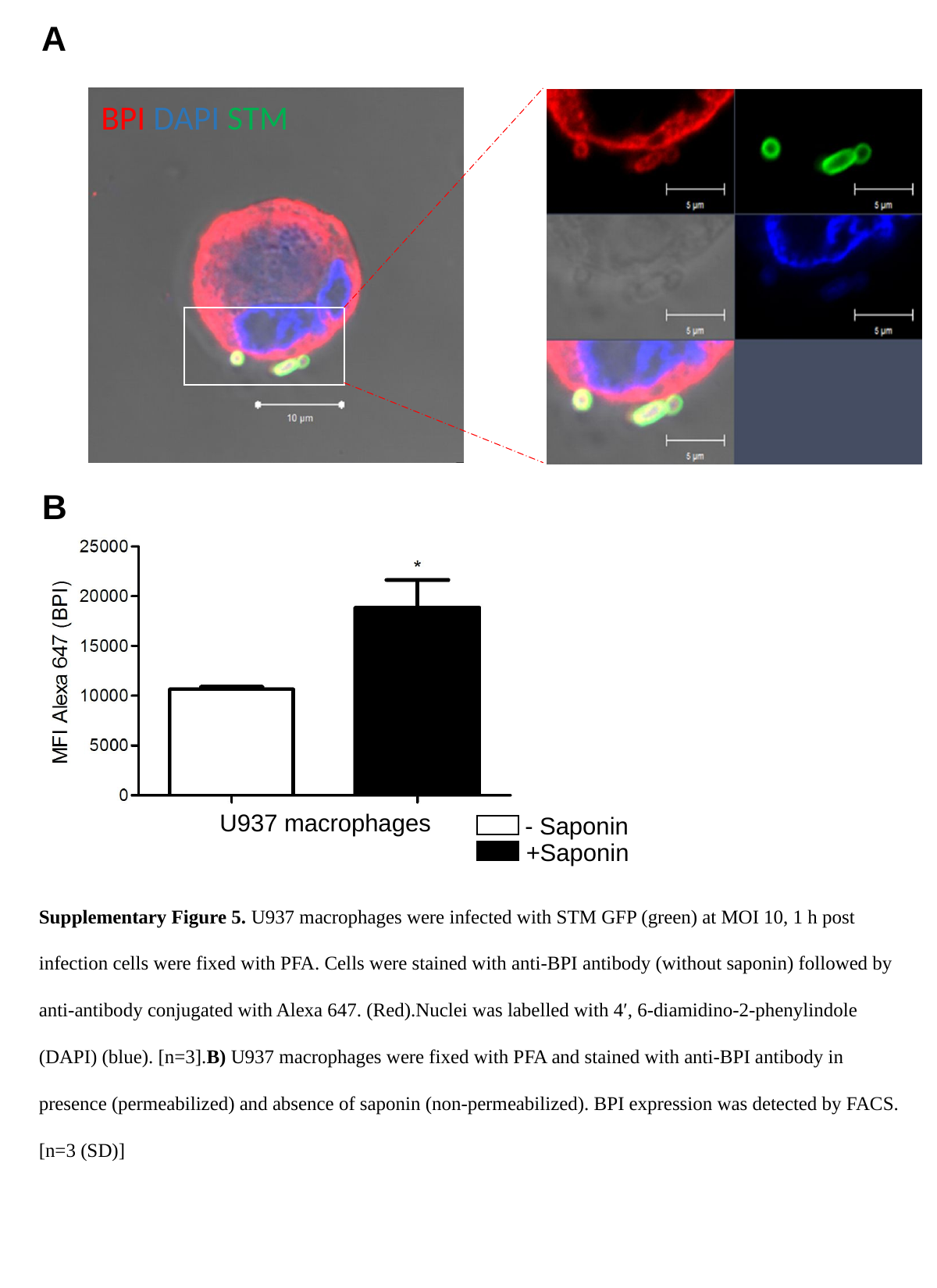

A
BPI DAPI STM
B
U937 macrophages
- Saponin
+Saponin
Supplementary Figure 5. U937 macrophages were infected with STM GFP (green) at MOI 10, 1 h post infection cells were fixed with PFA. Cells were stained with anti-BPI antibody (without saponin) followed by anti-antibody conjugated with Alexa 647. (Red).Nuclei was labelled with 4′, 6-diamidino-2-phenylindole (DAPI) (blue). [n=3].B) U937 macrophages were fixed with PFA and stained with anti-BPI antibody in presence (permeabilized) and absence of saponin (non-permeabilized). BPI expression was detected by FACS. [n=3 (SD)]

## Slide 4
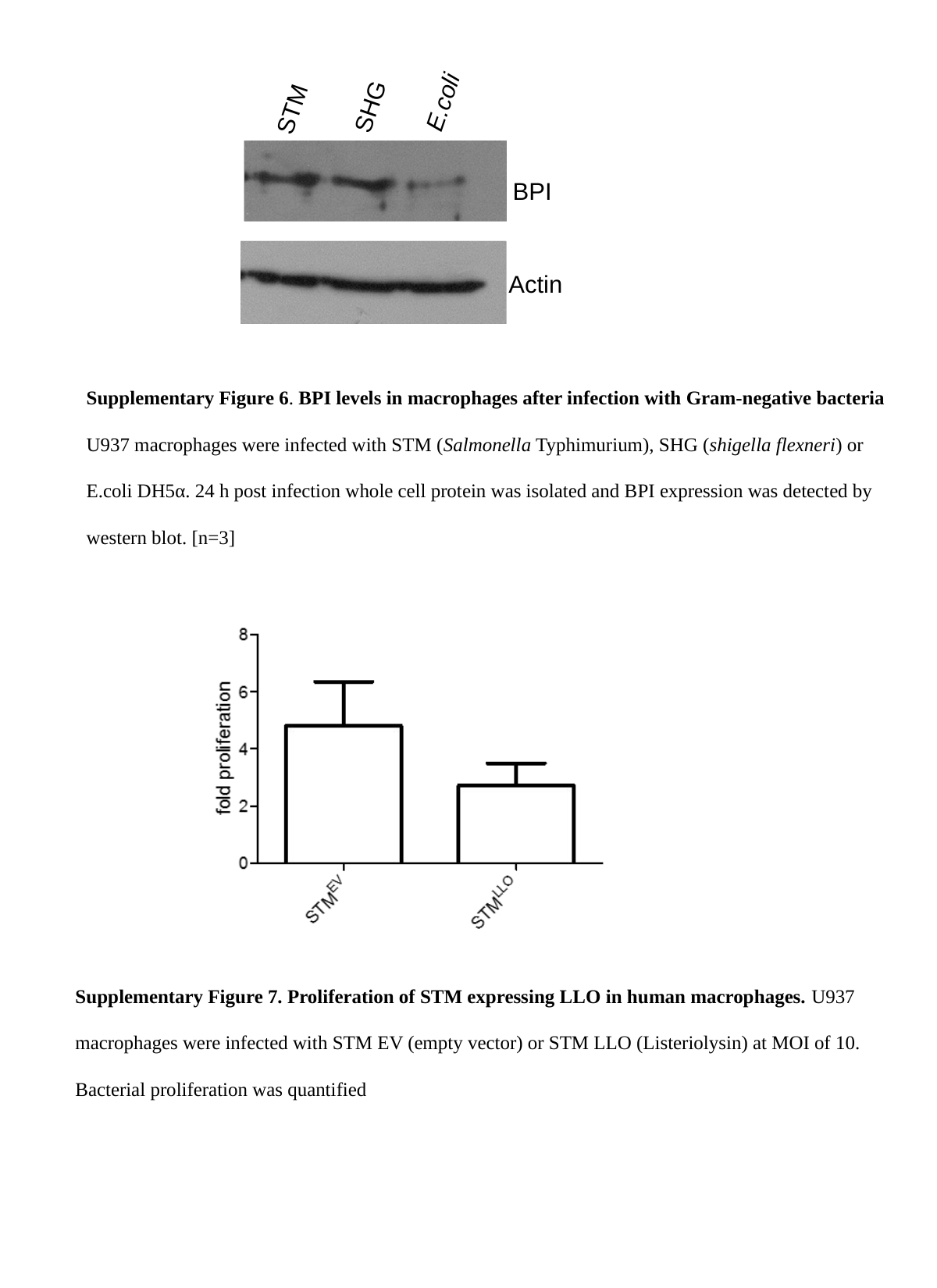

E.coli
SHG
STM
BPI
Actin
Supplementary Figure 6. BPI levels in macrophages after infection with Gram-negative bacteria U937 macrophages were infected with STM (Salmonella Typhimurium), SHG (shigella flexneri) or E.coli DH5α. 24 h post infection whole cell protein was isolated and BPI expression was detected by western blot. [n=3]
Supplementary Figure 7. Proliferation of STM expressing LLO in human macrophages. U937 macrophages were infected with STM EV (empty vector) or STM LLO (Listeriolysin) at MOI of 10. Bacterial proliferation was quantified
